# Invasive Acinetobacter baumannii ABC141 strain relies on the twin-arginine translocation export system for adhesion to host cells

**DOI:** 10.1099/mic.0.001630

**Published:** 2025-12-09

**Authors:** Charline Debruyne, Landon Hodge, Karsten Hokamp, Carsten Kröger, Anna S. Ershova, Suzana P. Salcedo

**Affiliations:** 1 Department of Pathobiological Sciences, School of Veterinary Medicine, University of Wisconsin-Madison, Madison, WI, USA; 2 Laboratory of Molecular Microbiology and Structural Biochemistry, Centre National de la Recherche Scientiﬁque UMR5086, Université de Lyon, Lyon, France; 3 Department of Genetics, School of Genetics & Microbiology, Smurfit Institute of Genetics, Trinity College Dublin, Dublin, Ireland; 4 Department of Microbiology, School of Genetics and Microbiology, Moyne Institute of Preventive Medicine, Trinity College Dublin, Dublin, Ireland

**Keywords:** ABC141, *Acinetobacter baumannii*, adhesion, twin-arginine translocation (Tat) system

## Abstract

*Acinetobacter baumannii* is associated with severe hospital-acquired, multi-drug-resistant infections worldwide, causing significant mortality and morbidity in intensive care patients or those under prolonged hospitalization. Multiple studies have recently shown that a proportion of circulating clinical isolates establish a transient multiplication niche inside phagocytic and non-phagocytic eukaryotic cells. We have previously demonstrated that the *A. baumannii* ABC141 strain invades human endothelial and epithelial cells, where it efficiently multiplies without induction of cytotoxicity. Here, we show that ABC141 adhesion, invasion and intracellular multiplication depend on the growth stage, being most efficient in the exponential growth phase. To define the gene expression signature most favourable to an intracellular lifestyle, a transcriptomic comparison was carried out between exponentially grown ABC141 and cultures in the stationary phase. Although most of the pathways identified reflected growth-related metabolic changes, we observed an up-regulation of the twin-arginine translocation (Tat) export system. Analysis of a mutant strain lacking the *tatABC* operon revealed that this export system is required only for adhesion to host cells, but not for invasion or intracellular multiplication. These data highlight a new role for the Tat export pathway in *A. baumannii* pathogenesis.

## Data Availability

Sequencing data are available at GenBank (PRJNA1255660) and Geo (GSE295948). Sequences of strains are available in Table S2 (available in the online Supplementary Material).

## Introduction

*Acinetobacter baumannii* is a major nosocomial pathogen causing significant morbidity and mortality in clinics worldwide, mainly due to its high rates of multi-drug resistance [1]. *A. baumannii* is most problematic in patients in intensive care or those with prolonged hospitalization, causing severe ventilator-associated pneumonia, septicaemia and burn and wound infections [2]. Traditionally, *A. baumannii* was regarded as an extracellular pathogen, but, as with many other bacteria, an intracellular stage has been identified. Multiple recent clinical isolates have been shown to invade non-phagocytic cells and to survive and multiply inside phagocytic and non-phagocytic cells [3,5]. This intracellular niche was observed *in vivo* in both urinary tract epithelial cells and lung macrophages [3,6] and was associated with enhanced dissemination in a murine model of urinary tract infection [6]. The intracellular stage may significantly impact the ability of clinicians to treat patients and may contribute to enhanced persistence within the host.

We recently identified *A. baumannii* strain ABC141 as invasive and able to multiply inside human epithelial and endothelial cells and keratinocytes [4]. Yet, the mechanisms enabling ABC141 to establish this niche remain unknown. In this study, we investigated the impact of the growth stage of ABC141 on the infection process, mapped the associated gene expression profiles and highlighted the role of the twin-arginine translocation (Tat) export system in its adhesion to both epithelial and endothelial cells.

## Methods

### Cell culture

American Type Culture Collection (ATCC) A549 (human epithelial lung cell line) and ATCC EA.hy 926 (human endothelial somatic cell line) were purchased from ATCC and grown in Dulbecco’s Modified Eagle Medium (DMEM) supplemented with 1% l-glutamine and 10% fetal calf serum (DMEMc) at 37 °C with a 5% CO_2_ atmosphere. Regular *Mycoplasma* tests were carried out to ensure the cells were negative.

### Bacterial strains and culture conditions

The *A. baumannii* ABC141 clinical isolate was obtained from the University of Cologne [4]. The mutant strain, ABC141Δ*tatABC,* was obtained following the protocol from [7]. Briefly, 2 kb upstream and downstream from the *tatABC* operon in genomic DNA of ABC141 were amplified by PCR with the high-fidelity polymerase VeriFi, using primers with overhangs for the cassette from the pMHL-2 plasmid (Table 1). This cassette contains the apramycin-resistance gene *aac* and the sucrose-susceptibility gene *sacB*. The strain ABC141 was transformed with an assembled PCR product of ~8 kb. Colonies were selected on Lennox broth (LB) agar (20 g l^−1^ LB and 15 g l^−1^ agar) supplemented with 30 µg ml^−1^ of apramycin, and susceptibility to sucrose was verified on LB agar without sodium chloride (NaCl), with 20% (w/v) sucrose. The ABC141 *tatABC::sacB_aac* was then naturally transformed with a chimeric PCR product composed of the 2 kb upstream and the 2 kb downstream of the *tatABC* operon, and mutants were selected on LB agar without NaCl, with 20% (w/v) sucrose; susceptibility to apramycin was then verified. The ABC141Δ*tatABC* mutant was verified using colony PCR and Sanger sequencing.

**Table 1.** Primers used in this study

Primer name	Sequence (5′−3′)
P1_tatABC_FwA	ACACGTAACATAGCTCACG
P2_tatABC_RvA	GGCCCAATTCGCCCTATAGTGAGTCGATCAACAGGTTGCCATGAC
P3_tatABC_FwB	GGGTTTGCTCGGGTCGGTGGCATATGCAATAAACTGTCTTCAAAGTGAC
P4_tatABC_RvB	CCGGTGTAGATAAAATACGG
P7_tatABC_Rv	GCTTTGTAGATTAAAAAGCGTTCATCAACAGGTTGCCATGAC
P8_tatABC_Fw	GAACGCTTTTTAATCTACAAAGC
P5_pMHL2_tatABC_Up	CGACTCACTATAGGGCGAATTGGGCCGCTTTCCAGTCGGGAAACCTG
P6_pMHL2_tatABC_Down	CATATGCCACCGACCCGAGCAAACCCCGCCAGGGTTTTCCCAGTCACGAC
P7_CD160_tat-Fw-promoter	GGACTTATCATCCAACCTGTGCAAAGCGCCAATAAGCGGTCTTTG
P8_CD162_tat-Rv-R3	CTCTGTGTCGCAATGGTGGATGACTTATTCAGCAGAATGGGTTTTTCTTTTCTC
P9_FwApraK7(R3)	CAGTAGAGTTAACTTATTTCCCAAGACAGGTTGGATGATAAGTCCCCG
P10_CD163_ApraK7-Rv-tat	CAAAGACCGCTTATTGGCGCTTTGCACAGGTTGGATGATAAGTCCCCG
P11_CD139_R3-FwA_pUC	GATCCTCTAGAGTCGACCTGCAGGCATGgcatgcGAATATTAAACAGCTTTTAAAAATCACCTTCGGG
P12_CD138_R3-RvA	CTTGGGAAATAAGTTAACTCTACTGAACAAT
P13_CD102_R3-FwB	GTCATCCACCATTGCGACACAGAGATAAG
P14_CD140_R3-RvB_pUC	CCATGATTACGCCAAGCTTgcatgcCGATCAAAACTTTGCGACTTTAGGTCAGCTC

The complementation of the *tatABC* deletion mutant was performed by amplifying the *tatABC* operon with 504 bp upstream to amplify the promoter region by PCR. In parallel, 2 kb upstream and downstream of the insertion site and the *aac* gene encoding apramycin resistance from the pMHL-2 plasmid were also amplified [7]. The fusion of all the fragments was possible by including overhangs in the primer design to allow Gibson assembly. Specifically, we used the NEBuilder^®^ HiFi DNA Assembly (New England Biolabs, E5520S) to fuse the fragments together in a pUC19 plasmid. The SphI-digested corresponding fragment was used to naturally transform the ABC141Δ*tatABC* mutant, which was then selected on LB agar supplemented with 30 µg ml^−1^ of apramycin. All primers are listed in Table 1.

The strains were grown on LB agar (pH 7.4) for 15 h at 37 °C. For overnight (ON) liquid cultures, a single colony was inoculated in LB (pH 7.4) and incubated for 15 h under shaking. All other cultures were obtained by diluting the overnight cultures to an optical density at 600 nm (OD_600_) of 0.1 and grown at 37 °C under shaking until an OD_600_ of 0.5, 0.8 and 1, or for 15 h to obtain an OD_600_ between 2.2 and 2.5, which we designate as the overnight culture.

### Human cell infections and adhesion assays

Human cells were grown in 24-well culture plates containing a coverslip at 1×10^5^ cells per well for the invasion and multiplication experiments. For the adhesion assays, to prevent attachment to the coverslip, we used a higher number of cells to obtain a confluent monolayer (2×10^5^ cells per well). In all experiments, cells were infected at a multiplicity of infection of 100 with the ABC141 strain diluted in prewarmed DMEMc. Plates were centrifuged at 400***g*** for 10 min and incubated at 37 °C with a 5% CO_2_ atmosphere. For the adhesion assay, after 1 h of incubation, cells were washed five times with PBS before being fixed with paraformaldehyde (3.7%) at room temperature (RT) for 15 min, followed by three PBS washes. For the invasion/multiplication assays, after 1 h of infection, cells were washed five times with PBS and incubated with DMEMc containing 50 µg ml^−1^ apramycin for 1 h. For the invasion assay, cells were fixed with paraformaldehyde 2 h post-infection. For the multiplication assay, 4 h post-infection, the DMEMc with apramycin was replaced with DMEMc for 22 h before fixation and washes.

### TRIzol RNA extraction

Total RNA from *A. baumannii* ABC141 was isolated essentially as described previously [8]. Briefly, *A. baumannii* ABC141 was grown overnight in LB at 37 °C under shaking, which corresponds to 15 h of growth and an OD_600_ between 2.2 and 2.5. This culture was diluted in LB to an OD_600_ of 0.1 before incubation under shaking at 37 °C until reaching an OD_600_ of 0.5. An ice-cold 5% (v/v) phenol–95% (v/v) ethanol ultra-pure solution was prepared, and 2/5 vol was added to 28 ml of the exponential or 15 ml of the ON cultures and incubated on ice for 1 h. Samples were pelleted by centrifugation at 3,220***g*** at 4 °C for 10 min, and cell pellets were subsequently dissolved in 1 ml ice-cold TRIzol and transferred into a Phase-Lock-Tube (VWR, #2302830). Four hundred µl of chloroform was added, and the solution was shaken without vortexing for 10 s, followed by a 3 min incubation at RT. Tubes were centrifuged at full speed for 15 min before the aqueous phase was transferred to a new 1.5 ml tube. RNA was precipitated by the addition of 450 µl of 2-propanol and incubation for 30 min at RT. After 30 min, the RNA was pelleted by centrifugation at full speed in a microcentrifuge, and the pellet was washed with 350 µl of ethanol (75%), incubated for 10 min and centrifuged for 10 min at full speed in a microcentrifuge. The wash step was repeated three times before discarding the remaining supernatant, air-drying the pellet and resuspending the RNA in 25 µl of RNase-free water, followed by a 5 min incubation at 65 °C at 900 r.p.m. with agitation. The RNA extractions were stored at −80 °C before assessing their quality using a 1/10 dilution of the extraction using the 4200 TapeStation (Agilent Technologies).

### Small RNA prediction

A blastN search was performed using 110 small RNAs identified in *A. baumannii* ATCC17978 [8] against the *A. baumannii* ABC141 genome (E-value <0.01). Hits with an alignment length exceeding 80% of the query length were annotated as potential small RNAs in *A. baumannii* ABC141.

### RNA sequencing and analysis

Total RNA was sent to the Core Unit Systems Medicine (SysMed) sequencing facility for RNA-sequencing (RNA-seq) (University of Würzburg, Germany), where DNase I digestion and rRNA depletion (RiboCop) were performed. Library for sequencing was prepared using the NEBNext Multiplex Small RNA Library Prep Kit. Sequencing was performed on the Illumina NextSeq-500 Mid Output KT v2.5 (150 cycles).

Bowtie2 v. 2.5.2, with the parameter -very-sensitive-local (to enable soft trimming and maximize the hit rate), was used to align sequencing reads to the reference genome. The gDNAx package v1.6.0 was used to assess genomic DNA contamination in the RNA-seq data and to filter out potential DNA reads [9]. FeatureCounts v2.0.6 was used to count the number of uniquely mapped reads. DESeq2 v1.44.0 was used to reveal genes differentially expressed between exponential and stationary phases (false discovery rate cutoff, 0.05) [10]. Gene Ontology (GO) term overrepresentation analysis was performed using the kegga method of the limma v3.60.6 R package [11].

### Immunolabeling

Once fixed, cells were permeabilized and blocked with a solution of PBS containing 0.1% and 2% BSA for 1 h at RT. To label bacteria, a mix of rabbit anti-ATCC 17978 (1 : 10,000), anti-C4 (1 : 1,000) and anti-AB5075 (1 : 1,000) from [4] was used. Mouse anti-LAMP1 H4A3 (1 : 200) from the Developmental Studies Hybridoma Bank, created by the Eunice Kennedy Shriver National Institute of Child Health and Human Development (NICHD) of the National Insitutes of Health (NIH) and maintained at the University of Iowa, was used to label *Acinetobacter*-containing vacuoles (ACVs) for the invasion and multiplication assays. Primary antibodies were diluted in the blocking solution and incubated for 2 h at RT. The coverslips were washed twice with the blocking solution. Cells were incubated for 1 h with the following secondary antibodies and dyes in the blocking solution: anti-rabbit Alexa Fluor 488 (1 : 1,000), anti-mouse Alexa Fluor 555 (1 : 1,000), phalloidin-Atto 565 (1 : 250) and DAPI nuclear dye (1 : 1,000). Final two washes were performed in the blocking solution, one wash in PBS and one in distilled water, before mounting the coverslips using ProLong™ Gold Antifade (Invitrogen).

### Counting the number of infected cells, intracellular bacteria and adhesion

For three independent experiments, the percentage of cells with intracellular bacteria was calculated at 2 h post-infection by counting the number of infected cells compared with the cell total in at least ten fields by microscopy. For the multiplication assay, intravacuolar bacteria were counted in three independent experiments at 2 and 24 h post-infection in at least ten different fields and 50 vacuoles. Finally, for the adhesion assay, ten fields for each condition were acquired by microscopy, in which the number of adherent bacteria was counted and normalized to the number of cells in each field. Statistical analysis was carried out on data from three independent experiments.

### Microscopy analyses

For image acquisition and analysis of the experiments comparing the OD_600_ and the different cell lines, a Zeiss LSM800 laser-scanning confocal microscope with a 63× oil-immersion objective was used. For the image acquisition of the WT versus *tat* mutant experiments, a Nikon Ti2E microscope with a 100× oil-immersion objective was used. Finally, images were analysed with Fiji [12] and assembled in Figure J [13].

### Quantification of bacterial length

Bacteria were grown to an OD_600_ of 0.5 and incubated with Hoechst (10 ng µl^−1^) for 30 min prior to imaging on LB agar (1%) pads using the Nikon Ti2E microscope with a 100× oil-immersion objective, capturing both phase-contrast and fluorescence images. Data were then analysed with MicrobeJ for quantification of the length of bacteria and frequency calculation [14].

### Signal peptide predictions

Signal peptides of protein sequences were predicted using SignalP 6.0 with the following settings: Organisms: Other, Output format: Long output and Model mode: Fast [15].

### Statistical tests

All datasets were tested for normality using the Shapiro–Wilkinson test, and a one-way ANOVA test with a Dunnett’s correction was used when comparing all conditions with a control condition, or Tukey’s correction for multiple comparisons. For two-dataset comparisons, a two-tailed unpaired t-test was used. All analyses were performed using GraphPad Prism 10.

## Results and discussion

### ABC141 grown to exponential phase shows enhanced bacterial adhesion, invasion and intracellular multiplication

**Fig. 1.**
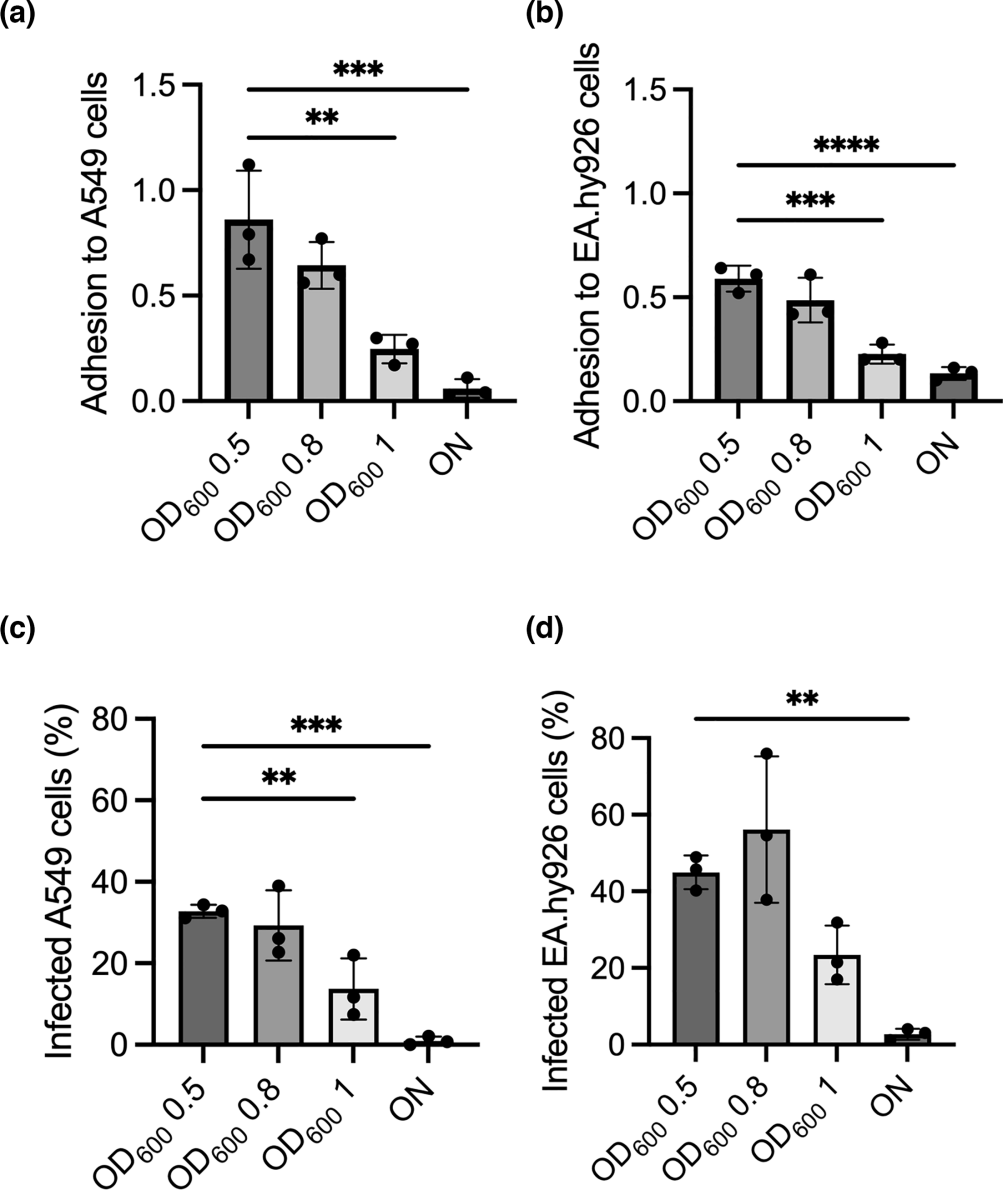
Adhesion and invasion of *A. baumannii* ABC141 in human epithelial and endothelial cells are reduced at stationary growth phase. (**a**) Quantification of the rate of adhesion to either A549 cells or (**b**) EA.hy926 endothelial cells after 1 h of infection and washes. Data correspond to the ratio of the number of adherent bacteria normalized to the number of cells observed per field by microscopy, presented as mean ± sd from three independent experiments. (**c**) Quantification of the percentage of A549 epithelial cells or (**d**) EA.hy926 endothelial cells infected after 2 h with inocula grown to OD_600_ 0.5, 0.8, 1 or 15 h culture with an OD_600_ between 2.2 and 2.5 (overnight). Data correspond to mean ± sd from three independent experiments quantified by microscopy. For all graphs, only significant comparisons are indicated in relation to OD_600_ 0.5, using a one-way ANOVA, with * indicating *P*<0.05; **P<0.01 and ***P<0.001.

We had previously shown that strain ABC141 of *A. baumannii* was invasive and capable of extended intracellular multiplication in comparison with other *A. baumannii* strains [4]. We hypothesized that a specific set of genes could be associated with these phenotypes in ABC141. We first compared the ability of ABC141 to adhere, invade and multiply in non-phagocytic cells when grown at different growth stages in LB. We used a microscopy-based approach because lysis of infected cells with detergents yielded variable results, potentially due to the impact of detergents on intracellular bacteria (Fig. S1, available in the online Supplementary Material), making c.f.u. counts unreliable. Starting from a common overnight culture, ABC141 was allowed to reach an optical density (OD_600_) of 0.8, which we previously showed to have a substantial invasion rate, with ~30% of cells infected at 1 h post-inoculation [4]. This condition was compared with cultures grown to an OD_600_ of 0.5, 1 or overnight (15 h). We did not test an OD_600_ lower than 0.5 to avoid having to concentrate cultures by centrifugation, which could impact the integrity of surface components, such as adhesins and pili. We observed that ABC141 adhesion to both epithelial and endothelial cells was steadily reduced with the increase in OD_600_ (Fig. 1a, b). The most marked decrease in adhesion was observed in overnight cultures. These results are not surprising, as the exponential growth stage has previously been shown to enhance the expression of several host factors important for cell adhesion in other *A. baumannii* strains, including the trimeric autotransporter adhesin (Ata) and pili [16,18].

To investigate the impact of each condition on bacterial invasion, we quantified the percentage of infected cells at 2 h post-infection, which includes a 1 h treatment with apramycin to kill extracellular bacteria. The quantification of the number of infected cells in the monolayer provides a readout of the ability of ABC141 to invade host cells. We did not count individual bacteria, as they likely multiply within the first 2 h post-internalization, which could artificially increase invasion rates. ABC141 internalization was steadily reduced with the increase in OD_600_, with a strong inhibition when the inoculums were prepared from the overnight cultures in both A549 epithelial cells (Fig. 1c) and endothelial cells (Fig. 1d). These results are consistent with the adhesion defects observed, suggesting that the genes expressed in the exponential phase are more permissive for ABC141 adhesion and invasion of host cells than those expressed in the stationary growth phase.

**Fig. 2.**
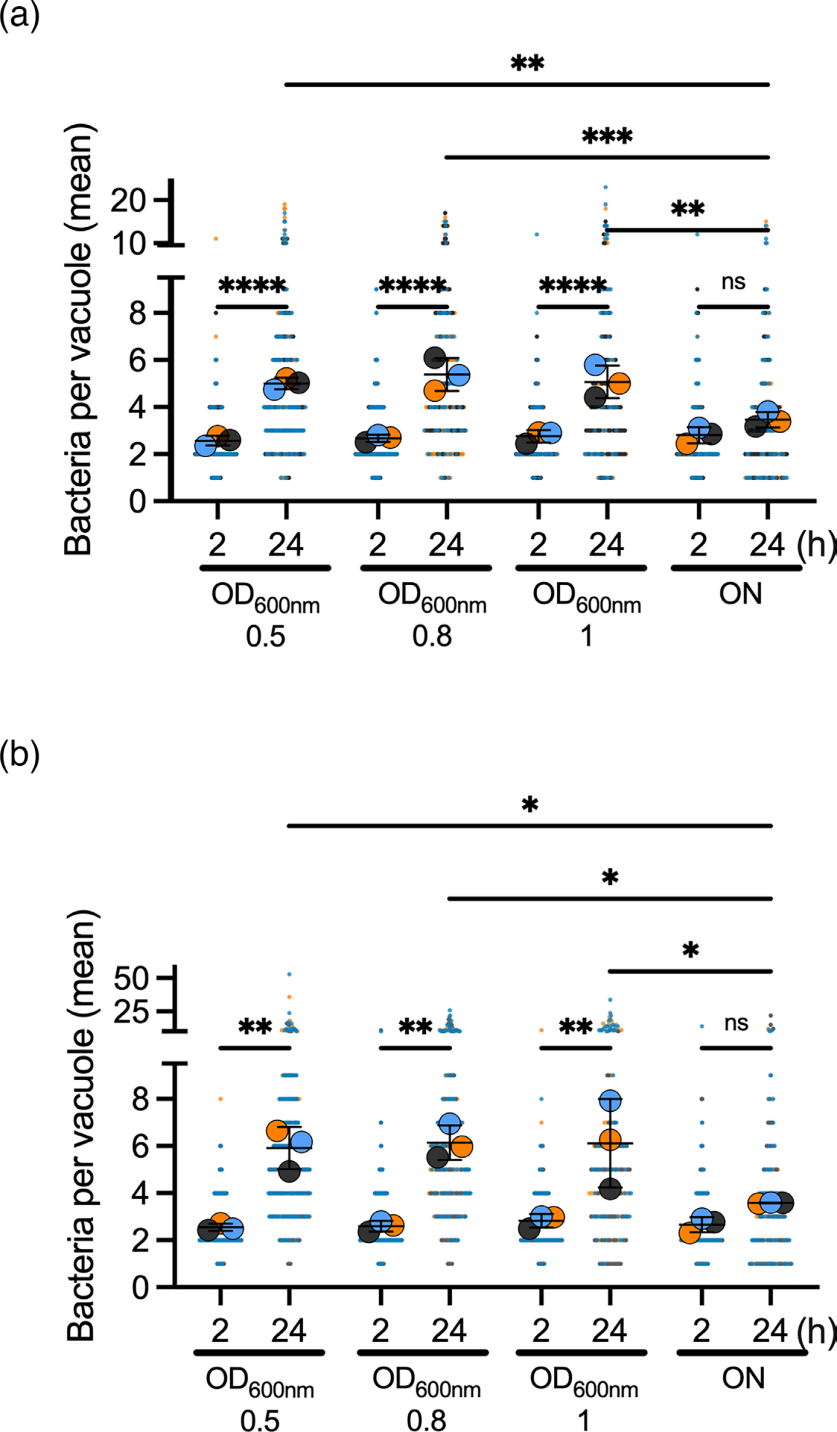
Intracellular multiplication of *A. baumannii* ABC141 in human epithelial and endothelial cells is reduced at stationary growth phase. Quantification of the number of intracellular bacteria per vacuole at 2 and 24 h post-infection in (**a**) A549 cells or (**b**) EAh.hy926 cells infected with ABC141 grown to OD_600_ values of 0.5, 0.8, 1 or 15 h culture (overnight). Data correspond to mean ± sd from three independent experiments quantified by microscopy. Each vacuole counted is shown with the small dots, and each experiment is colour coded. For all graphs, only relevant comparisons are indicated, using a one-way ANOVA, with * indicating *P*<0.05; **P<0.01, ***P<0.001 and ****P<0.0001. ns, non-significant.

Next, we estimated the ability of ABC141 in each condition to multiply inside cells by counting the number of bacteria per vacuole at 2 and 24 h. This approach enables us to assess intracellular multiplication at the single-cell level, independently of the reduced invasion phenotype. We found that infecting cells with ABC141 grown to the stationary phase resulted in almost absent intracellular multiplication in both cell types (Fig. 2a, b). These data indicate that the growth stage impacts the expression of bacterial factors that favour the establishment of a replicative intracellular niche for *A. baumannii* ABC141. It is possible that stationary-phase bacteria are not expressing the virulence determinants required for the intracellular trafficking of the ACV. Alternatively, these bacteria potentially induce a different entry mechanism that may lead to an ACV incompatible with ABC141 multiplication.

### Gene expression profiling of *A. baumannii* ABC141

**Fig. 3.**
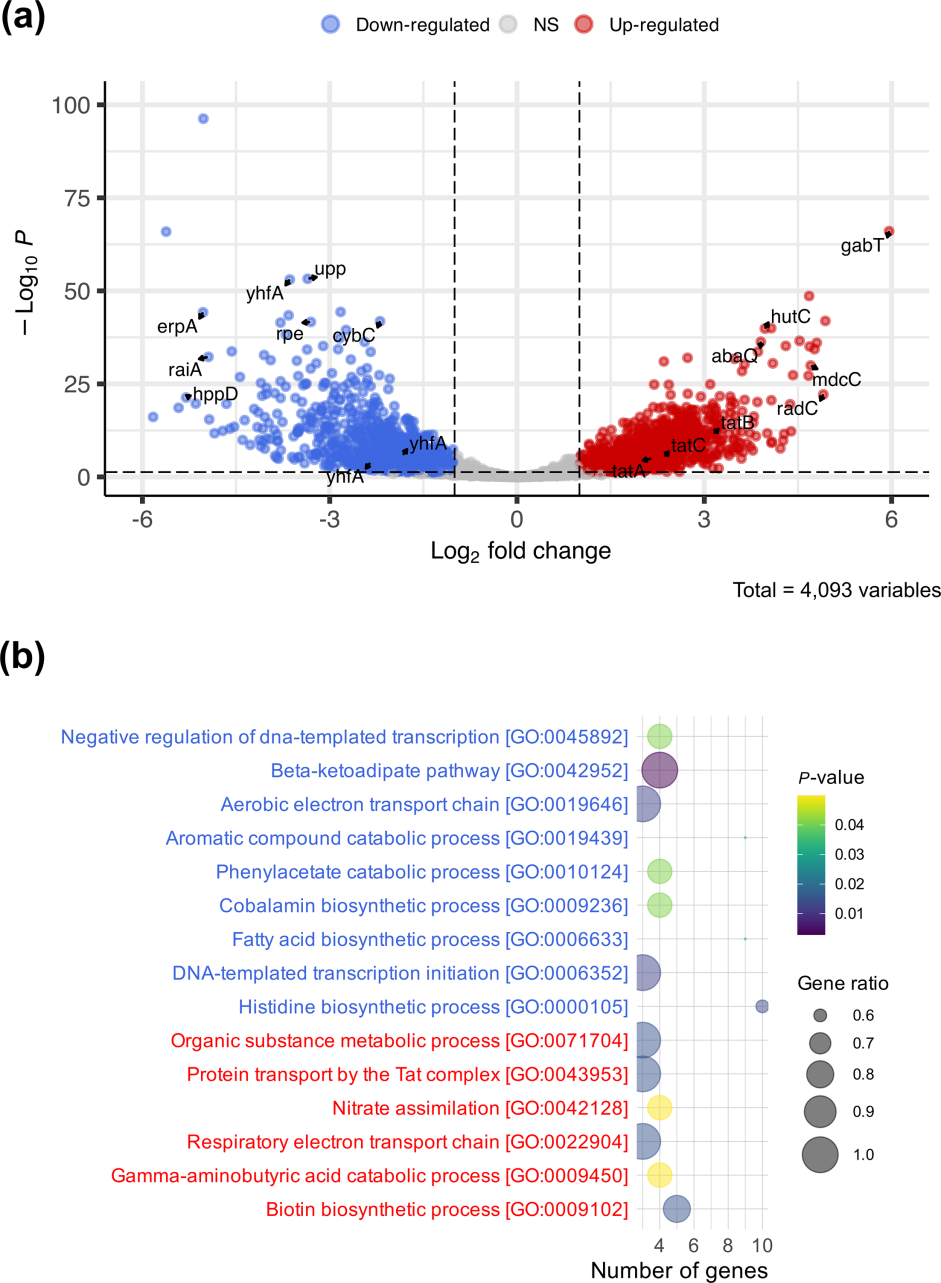
Differential gene expression and GO enrichment analysis of *A. baumannii* ABC141. (**a**) Volcano plot showing differential gene expression of *A. baumannii* ABC141 grown to the exponential versus the stationary phase. The *x*-axis represents the log₂ fold change, and the *y*-axis represents the –log₁₀ adjusted *P*-value. Genes with a log₂ fold change ≤–1 and an adjusted *P*-value <0.05 are considered down-regulated (highlighted in blue), while genes with a log₂ fold change ≥1 and an adjusted *P*-value <0.05 are considered up-regulated (highlighted in red). All other genes are considered non-significantly changed (NS). (**b**) GO enrichment analysis of genes associated with up-regulation and down-regulation. Enriched GO terms in the Biological Process category are shown with *P*-value <0.05. Terms associated with up-regulated genes are labelled in red font, and those associated with down-regulated genes are labelled in blue font.

To gain insight into what might cause the differences in cell infections, we carried out an RNA-seq experiment to compare the global gene expression profiles of cells grown to the exponential (OD_600_=0.5) and stationary phases (cells grown overnight; 15 h) in LB (three biological replicates; Fig. S2). We observed that 779 genes were ≥3-fold up-regulated in the exponential phase compared with the stationary phase, while 736 genes were ≥3-fold down-regulated (Fig. 3a, Table S1). We also noted that a larger number of small RNAs were expressed at a higher level in stationary phase, which is similar to findings in other Gram-negative pathogens because small RNAs are often involved in managing stress during the stationary phase [19]. A browser for the RNA-seq data can be found at: https://bioinf.gen.tcd.ie/jbrowse2/?config=kroegerlab%2FABC141%2Fconfig.json. A GO analysis showed differential expression of multiple biological pathways (Fig. 3b). As the differential expression of metabolic pathways could primarily reflect different nutrient availability, we focused on processes that might be more directly involved in adhesion and invasion of eukaryotic cells, such as protein export and secretion systems. One of the protein export systems up-regulated in the exponential phase compared with the stationary phase was the Tat export pathway, consisting of *tatA*, *tatB* and *tatC* (4.4–9.6-fold up-regulated in the exponential phase; *P*<0.001; Fig. 3b and Table S1), which has not been studied in *A. baumannii*. The Tat export pathway has been implicated in the virulence of many Gram-negative pathogens, notably in *Pseudomonas aeruginosa* and *Escherichia coli* [20,21] (reviewed in [22]). Therefore, we next focused on the impact of the Tat translocase on cell adhesion, invasion and intracellular stage of infection.

### The Tat export system is required for host cell adhesion but not for invasion or intracellular multiplication

**Fig. 4.**
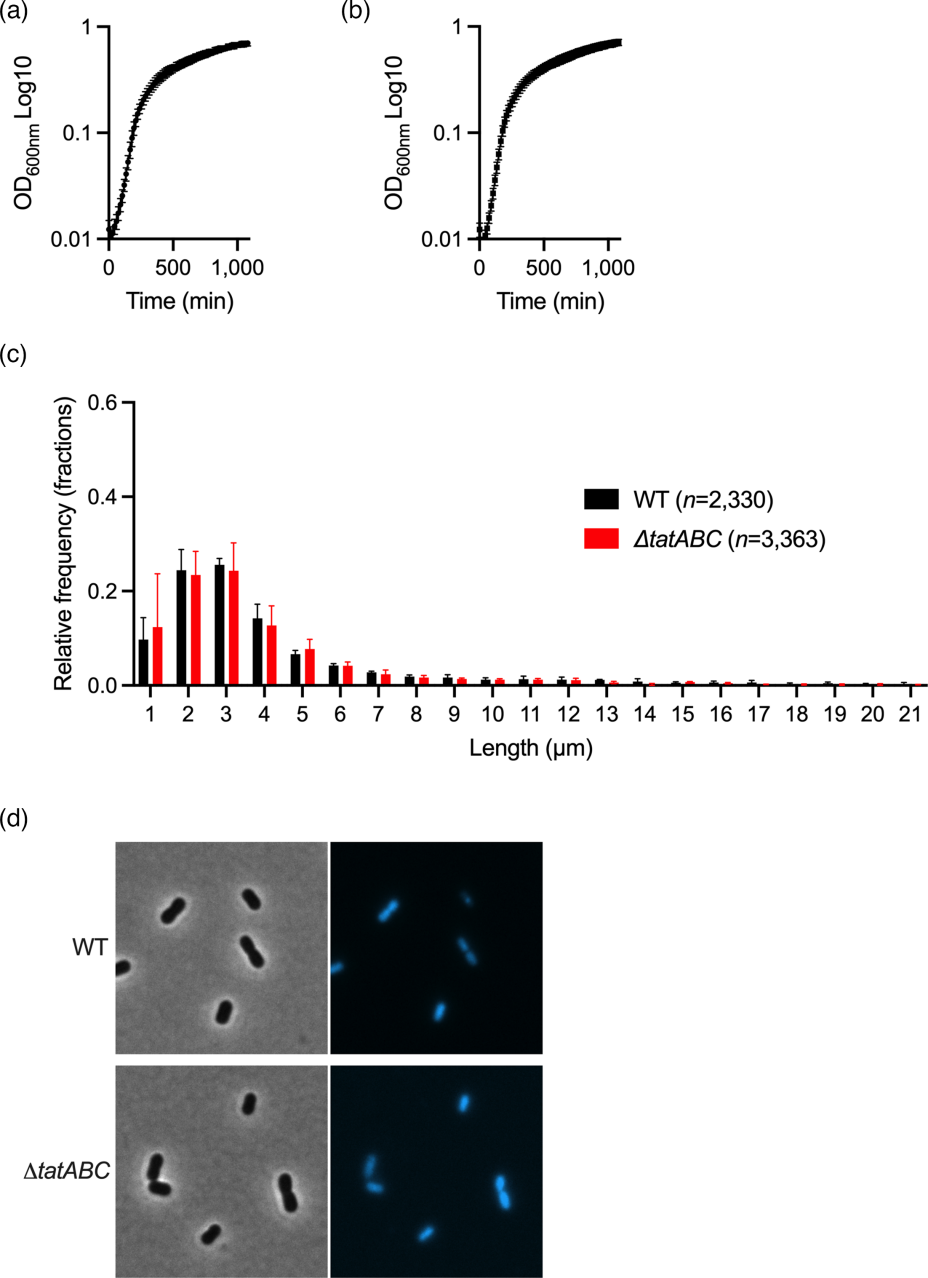
Deletion of *tatABC* does not significantly impact bacterial morphology and growth. (**a**) Bacterial growth curve for the ABC141 WT and (**b**) the mutant lacking the *tatABC* operon (Δ*tatABC*). Data correspond to mean ± sd (*n*=3). (**c**) Relative frequency distribution of the lengths of WT and Δ*tatABC* mutants analysed by MicrobeJ for three independent cultures. The total number of bacteria analysed is shown. (**d**) Representative phase-contrast and fluorescence images of WT and Δ*tatABC* labelled with Hoechst (cyan).

As the Tat system is known to export important proteins implicated in cell division [23], we first assessed the impact of deleting the TatABC operon on bacterial growth and morphology. Validation of the mutant was performed by sequencing (Fig. S3). The mutant strains grew as efficiently as the WT (Fig. 4a, b). Microscopy analysis of the length of the bacteria did not reveal any significant cell division defects between the two strains (Fig. 4c), which were morphologically indistinguishable by microscopy (Fig. 4d).

**Fig. 5.**
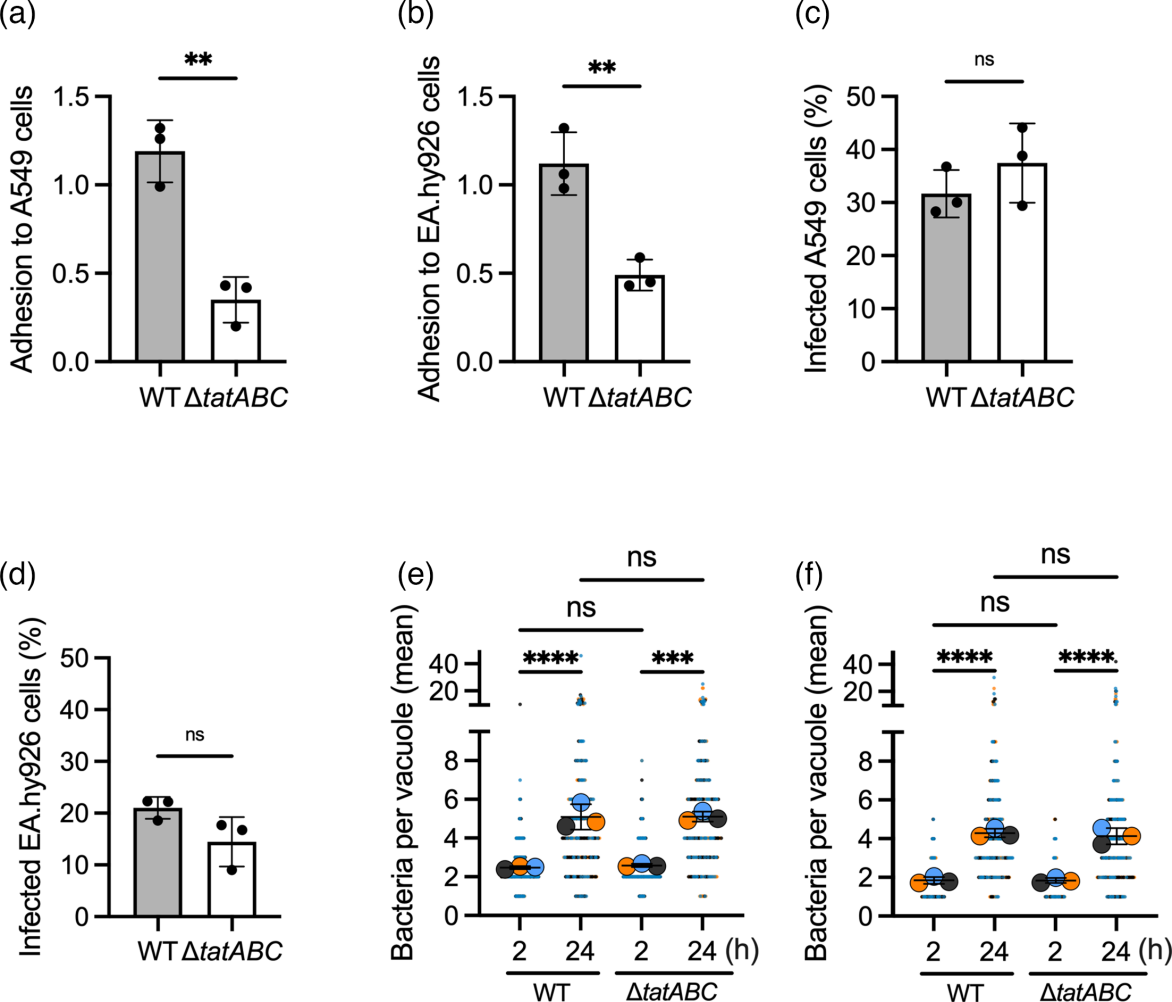
The Tat export system is implicated in host cell adhesion but not in invasion or intracellular multiplication of human epithelial and endothelial cells. (**a**) Quantification of the rate of adhesion to A549 cells or (**b**) EA.hy926 cells after 1 h of infection and washes. Data correspond to the ratio of the number of adherent bacteria normalized to the number of cells observed per field by microscopy, presented as mean ± sd from three independent experiments. (**c**) Quantification of the percentage of infected A549 cells or (**d**) EA.hy926 cells at 2 h post-infection with either the WT ABC141 *A. baumannii* or its corresponding mutant lacking the full *tatABC* operon (Δ*tatABC*). Data correspond to mean ± sd from three independent experiments quantified by microscopy. (**e**) Quantification of the number of intracellular bacteria per vacuole at 2 and 24 h post-infection in A549 cells or (**f**) EA.hy926 cells infected with WT ABC141 or the mutant lacking the *tatABC* operon. Data correspond to mean ± sd from three independent experiments quantified by microscopy. Each vacuole counted is shown as small dots, and each experiment is colour coded. Only relevant comparisons are indicated, performed using a one-way ANOVA, with **P<0.01, ***P<0.001 and ****P<0.0001. ns, non-significant.

**Fig. 6.**
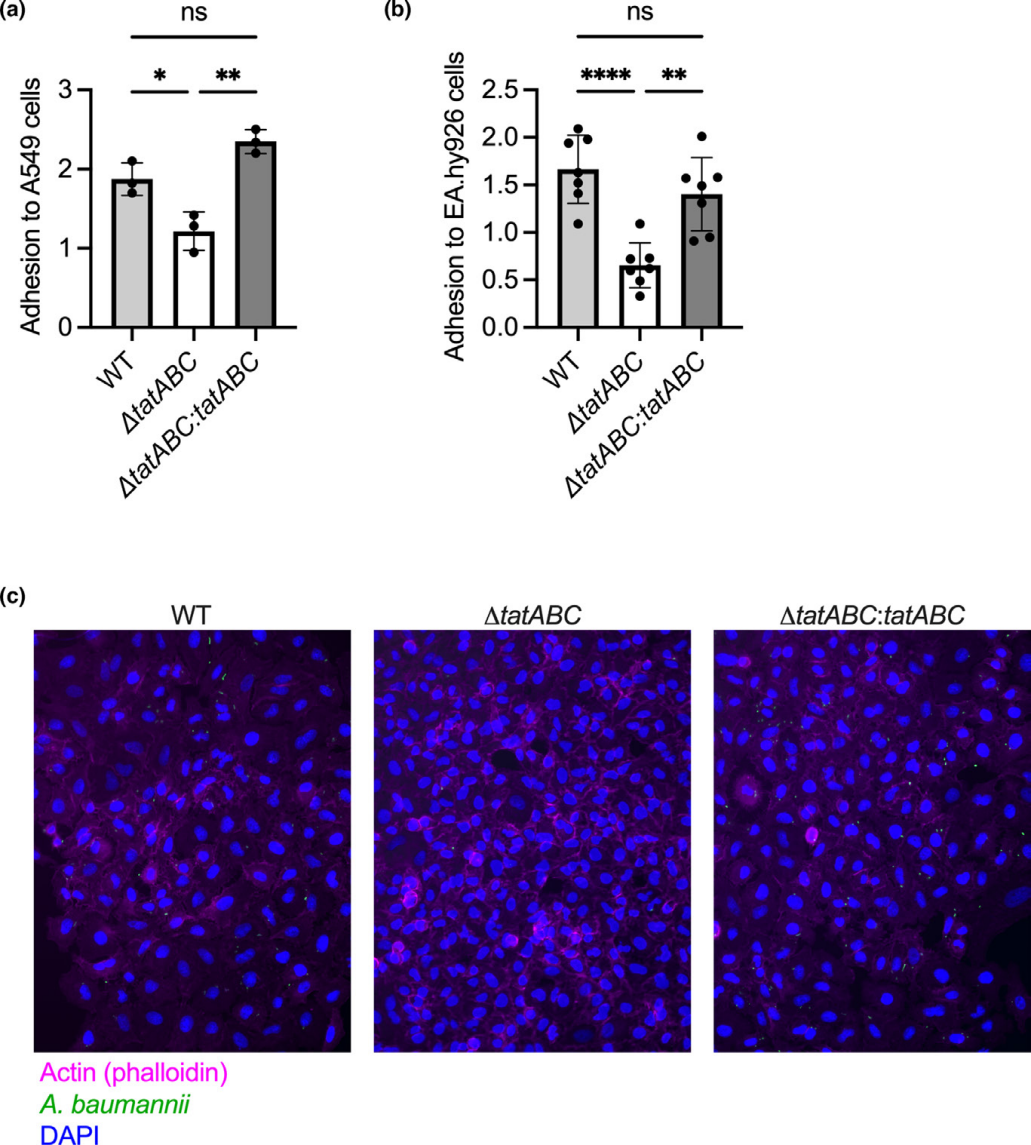
The defect of the Δ*tatABC* mutant adhesion is specifically due to the lack of the *tatABC* operon. (**a**) Quantification of the rate of adhesion of either the WT ABC141 *A. baumannii* or its corresponding mutant lacking the full *tatABC* operon (Δ*tatABC*) and the complemented strain (Δ*tatABC:tatABC*) to A549 or (**b**) EA.hy926 cells after 1 h of infection and washes. Data correspond to the ratio of the number of adherent bacteria normalized to the number of cells observed per field by microscopy, presented as mean ± sd from three independent experiments. A one-way ANOVA, with *P=0.0172, **P<0.01, ***P<0.001 and ****P<0.0001. (**c**) Representative immunofluorescence images of A549 cells showing adherent WT ABC141 *A. baumannii* (left panel), the mutant lacking the full *tatABC* operon (Δ*tatABC*; middle panel) and the complemented strain (Δ*tatABC:tatABC;* right panel). Bacteria were visualized with an anti-*Acinetobacter* antibody (green), the nuclei with DAPI (blue) and the actin cytoskeleton with phalloidin (magenta). ns, non-significant.

To assess the Tat system in the context of *A. baumannii* adhesion, invasion and intracellular multiplication, the *tatABC* operon was deleted in *A. baumannii* ABC141. Infection of either A549 or EA.hy926 cells with either the WT or a mutant strain lacking the entire *tatABC* operon revealed a clear inhibition of adhesion (Fig. 5a, b), but without significantly impacting invasion (Fig. 5c, d) or intracellular multiplication rates (Fig. 5e, f). These results suggest that the first step of the ABC141 interaction with non-phagocytic cells requires a functional Tat export system. To unequivocally attribute the observed effects to the loss of Tat function, we complemented the Δ*tatABC* by expressing the *tatABC* operon in the chromosome under the control of its native promoter. The adhesion defect of the Δ*tatABC* was abolished in the complemented strain in both epithelial and endothelial cells (Fig. 6a, b). Representative images of the phenotypes in A549 cells are shown in Fig. 6c.

It is intriguing that the invasion was not significantly impacted, suggesting that there is an important bottleneck at this stage of the infection, and not all adherent bacteria can be internalized into host cells or actively invade them. Further work is needed to understand the molecular mechanisms involved, notably by identifying Tat substrates and the host receptors mediating this process and specific ABC141 adhesins.

An important virulence factor of *A. baumannii* is its type 2 secretion system (T2SS), which relies on the general secretion (Sec) or Tat export systems for protein export across the inner membrane [24,25]. One known *A. baumannii* T2SS substrate, InvL, has been implicated in adhesion to host cells, but it was not shown to be exported via the Tat system and does not possess a Tat secretion signal in its sequence [26]. Indeed, none of the other known T2SS substrates have been reported to have a Tat secretion signal. Therefore, other yet-to-be-identified adhesins exported by the Tat system may play a role in ABC141 attachment and adhesion to host cells. A role for the Tat export pathway in adhesion has been demonstrated for *Achromobacter xylosoxidans* infection of bronchial epithelial cells, but no substrate was identified [27]. Analysis of the ABC141 genome for Tat secretion signals (SignalP6.0 prediction) did not yield significant candidates (Supplementary Material 2).

It is possible that FimA, which is also highly up-regulated (25.6-fold) during the exponential growth phase compared with the stationary phase, is a major contributor to the initial steps of ABC141 infection of host cells. FimA is a major structural component of type 1 pili, required for host cell adhesion of other pathogens, such as uropathogenic *E. coli* [28]. Its assembly follows the chaperone–usher pathway, which is dependent on the Sec export system and independent of Tat [29]. Consistently, FimA has no canonical Tat secretion signal, as per SignalP 6.0 prediction. However, in haloarchaea, the Tat system has been shown to export components of thin, filamentous surface appendages that could contribute to adhesion to surfaces and bacteria–bacteria interactions [30]. These Tat-fimbriae, also called ‘tafi’, are composed of a core component called TafA, with some similarities to FimA. Further work is necessary to confirm the function of ABC141 FimA and to identify the Tat export system substrates implicated in host cell attachment and adhesion.

## Conclusion

Although the most efficient adhesion, invasion and intracellular multiplication of *A. baumannii* ABC141 were observed in cultures grown to the exponential phase, we have not identified a marked gene expression signature using RNA-seq in culture. However, we have found that the Tat system is required for adhesion to human epithelial and endothelial cells but is dispensable for invasion and intracellular multiplication. These results show a novel role for the Tat export pathway in ABC141 pathogenesis.

## Supplementary material

10.1099/mic.0.001630 Uncited Supplementary Material 1.

10.1099/mic.0.001630 Uncited Supplementary Material 2.

10.1099/mic.0.001630 Uncited Table S1.

10.1099/mic.0.001630 Uncited Table S2.

## Abbreviations

ACV: *Acinetobacter*-containing vacuole
Ata: autotransporter adhesin
ATCC: American Type Culture Collection
DMEM: Dulbecco’s Modified Eagle Medium
DMEMc: supplemented Dulbecco’s Modified Eagle Medium
GO: Gene Ontology
LB: Lennox broth
OD_600_: optical density at 600 nm
RNA-seq: RNA-sequencing
RT: room temperature
Sec: general secretion
Tat: twin-arginine translocation
T2SS: type 2 secretion system
