# Supplementary material for: Invasive Acinetobacter baumannii ABC141 strain relies on the twin-arginine translocation export system for adhesion to host cells
Source: Microbiology (Reading). 2025 Dec 9;171(12):001630. doi: 10.1099/mic.0.001630 (PMC12687288; doi:10.1099/mic.0.001630)
Supplement: Uncited Supplementary Material 1. [file mic-171-01630-s001.pdf]

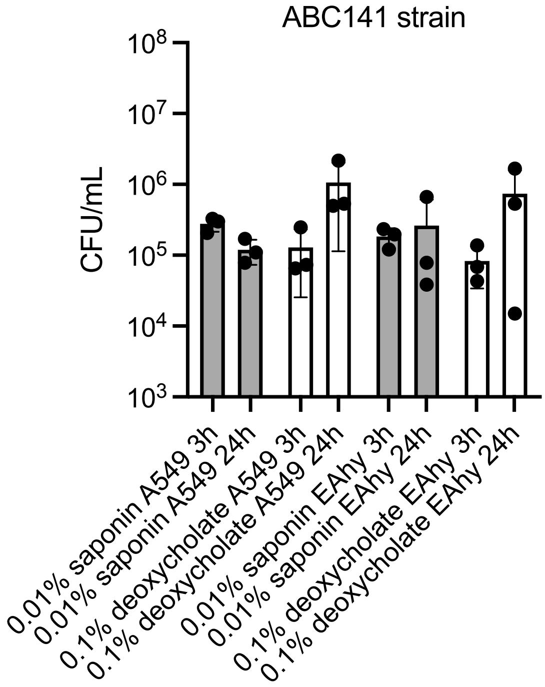

Supplementary Figure 1. Lysis of infected cells with different detergents for bacterial CFU enumeration. Quantification of the bacterial CFU counts from A549 and EA.hy cells infected for 3 or 24h with ABC141 and lysed with either 0.01% (reviewer suggestion, grey bars) or 0.1% deoxycholate (commonly used in our lab). Data correspond to 3 independent experiments and are presented as means ± SD.

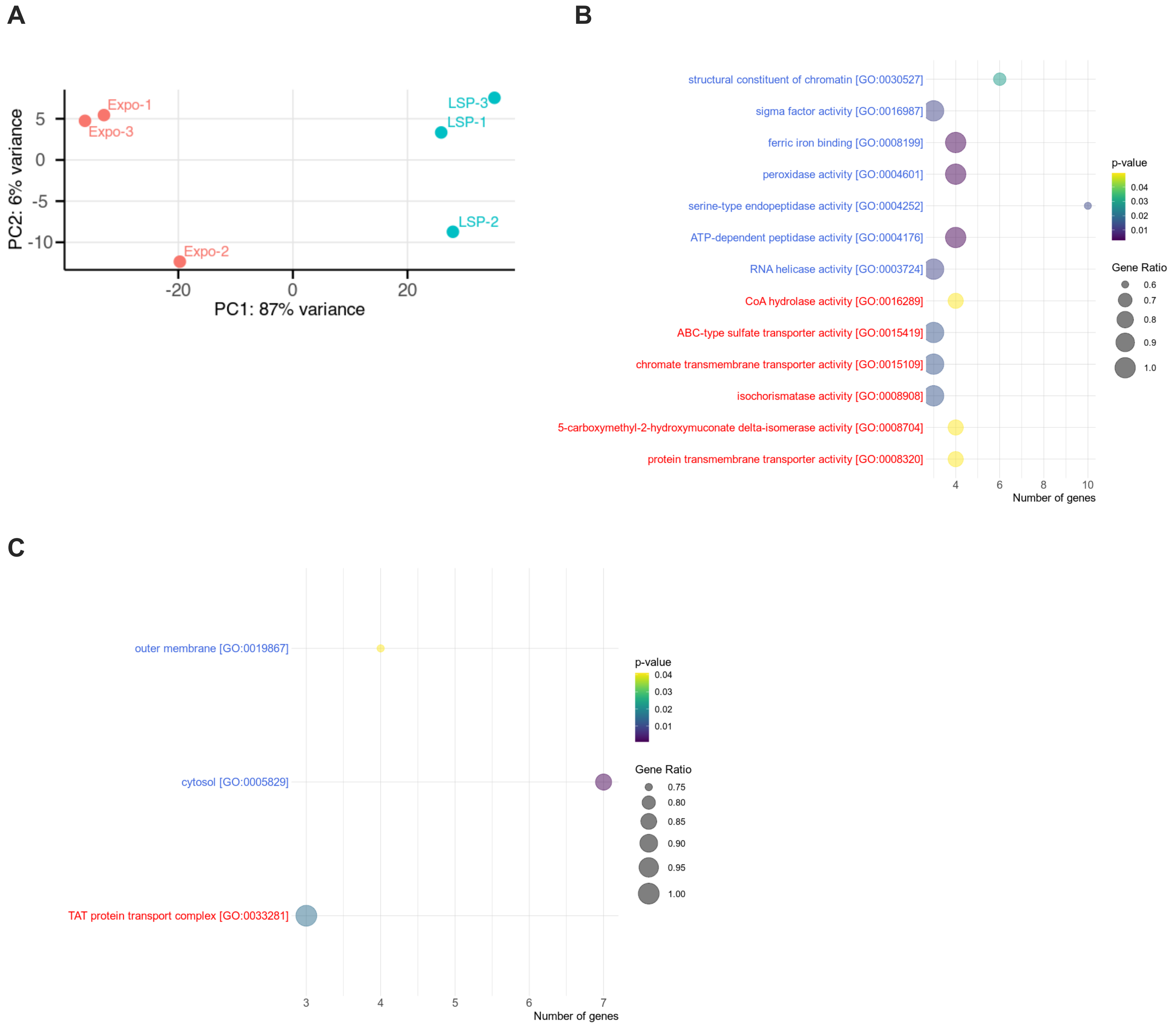

Supplementary Figure 2. Principal component analysis and GO enrichment of differentially expressed genes. **(A)** Principal Component Analysis plot showing the variance in gene expression profiles between Exponential (ESP) and Late Stationary phase (LSP) samples. Each point represents a biological replicate, and the axes indicate the percentage of variance explained by the first two principal components (PC1: 87%, PC2: 6%). (**B**) Gene Ontology (GO) enrichment analysis of genes associated with upregulated and downregulated genes. Enriched GO terms in the Molecular Function category are shown (p-value < 0.05). (**C**) GO enrichment analysis of genes associated with upregulated and downregulated genes. Enriched GO terms in the Cellular Component category are shown (p-value < 0.05). Terms associated with upregulated genes are labeled in red font, and those associated with downregulated genes are labeled in blue font. GO analysis is performed using kegga function of limma (v3.60.6) package.

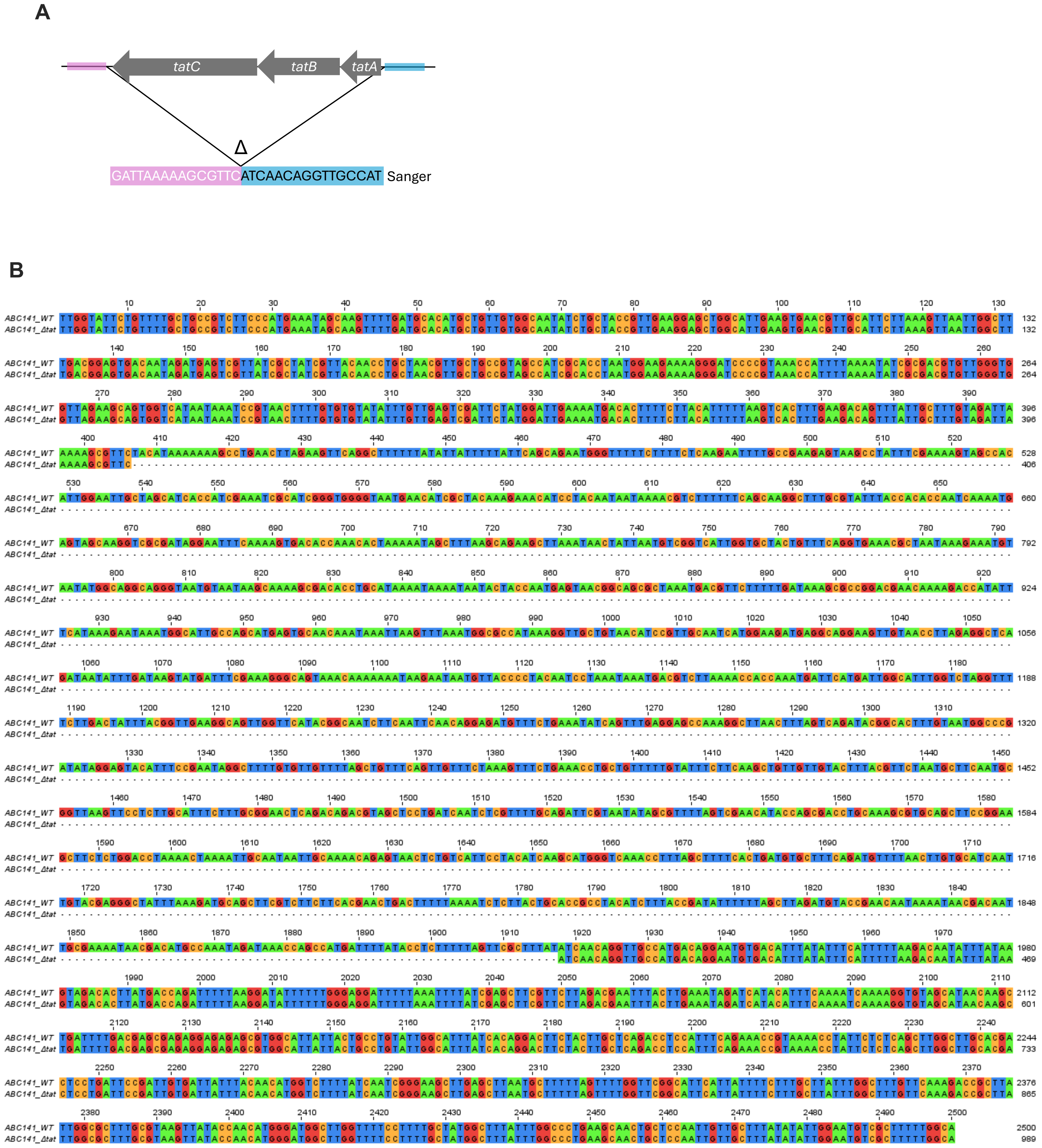

Supplementary Figure 3. Validation of gene deletion of the *tatABC* operon.
(A) Diagram of the deletion strategy. **(B)** Alignment of the Sanger sequences obtained for the wild-type and mutant deleted for the *tatABC* operon.
